# 1-Methyl-6-nitro-1*H*-benzimidazole

**DOI:** 10.1107/S1600536808005886

**Published:** 2008-03-05

**Authors:** Jan Lokaj, Viktor Kettmann, Tomáš Solčan, Svetozar Katuščák

**Affiliations:** a Faculty of Food and Chemical Technology, Slovak Technical University, Radlinskeho 9, SK-81237 Bratislava, Slovak Republic; b Faculty of Pharmacy, Comenius University, Odbojarov 10, SK-83232 Bratislava, Slovak Republic

## Abstract

The title compound, C_8_H_7_N_3_O_2_, a potential anti­tumour drug and an anti­oxidant agent, was studied in order to give more insight into structure–function relationships. The 1-methyl­benzimidazole unit of the mol­ecule was found to be exactly planar and the nitro group is inclined at an angle of 10.4 (2)° to the plane of the heterocycle. The bond lengths in the present derivative were analyzed in details and compared with those of the parent unsubstituted analogues in the Cambridge Structural Database. The results have shown that the additional nitro group is not involved in conjugation with the adjacent π-system and hence has no effect on the charge distribution of the heterocyclic ring.

## Related literature

For related literature on related crystal structures, see for example: Türktekin *et al.*, (2004 ▶) as retrieved from the Cambridge Structural Database (Version of 2007; Allen, 2002 ▶). For the synthesis, see: Ellis & Jones (1974 ▶). For the length of the pure C*sp* ^2^—N*sp* ^2^ single bond, see: Adler *et al.* (1976 ▶). For related literature on biological aspects of the benzimidazole derivatives in general, see: Alpan *et al.* (2007 ▶); Kettmann *et al.* (2004 ▶); Le *et al*. (2004 ▶); Nguyen *et al.* (2004 ▶); Statkova-Abeghe *et al.* (2005 ▶). Anti­oxidant properties of the compound are discussed by Hanus *et al.* (2004 ▶); Katuščák (2003 ▶).
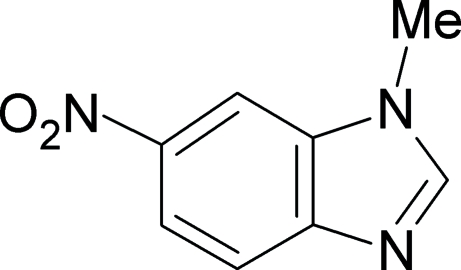


## Experimental

### 

#### Crystal data

C_8_H_7_N_3_O_2_
*M* *_r_* = 177.17
Orthorhombic, 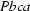

*a* = 12.852 (3) Å
*b* = 7.043 (2) Å
*c* = 17.690 (4) Å
*V* = 1601.2 (7) Å^3^
*Z* = 8
Mo *K*α radiation
μ = 0.11 mm^−1^
*T* = 296 (2) K
0.30 × 0.20 × 0.15 mm

#### Data collection

Siemens P4 diffractometer
Absorption correction: none
3027 measured reflections
2325 independent reflections
1493 reflections with *I* > 2σ(*I*)
*R*_int_ = 0.035
3 standard reflections every 97 reflections intensity decay: none

#### Refinement

*R*[*F* ^2^ > 2σ(*F* ^2^)] = 0.045
*wR*(*F* ^2^) = 0.130
*S* = 0.96
2325 reflections
119 parameters
H-atom parameters constrained
Δρ_max_ = 0.18 e Å^−3^
Δρ_min_ = −0.15 e Å^−3^

### 

Data collection: *XSCANS* (Siemens, 1991 ▶); cell refinement: *XSCANS*; data reduction: *XSCANS*; program(s) used to solve structure: *SHELXS97* (Sheldrick, 2008 ▶); program(s) used to refine structure: *SHELXL97* (Sheldrick, 2008 ▶); molecular graphics: *PLATON* (Spek, 2003 ▶); software used to prepare material for publication: *SHELXL97*.

## Supplementary Material

Crystal structure: contains datablocks global, I. DOI: 10.1107/S1600536808005886/nc2091sup1.cif

Structure factors: contains datablocks I. DOI: 10.1107/S1600536808005886/nc2091Isup2.hkl

Additional supplementary materials:  crystallographic information; 3D view; checkCIF report

## Figures and Tables

**Table 1:** Selected bond lengths (Å)

N1—C2	1.3571 (17)
N1—C8	1.3817 (17)
C2—N3	1.3107 (19)
N3—C9	1.3803 (18)
C4—C5	1.366 (2)
C4—C9	1.4001 (19)
C5—C6	1.4095 (19)
C6—C7	1.3840 (19)
C6—N4	1.4563 (18)
C7—C8	1.3775 (18)
C8—C9	1.4104 (17)

**Fig. 1.**
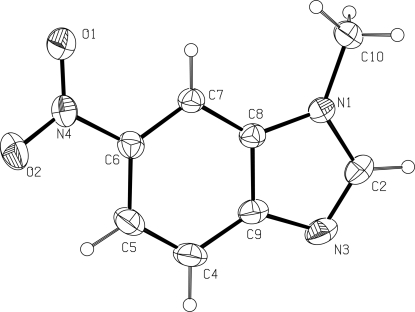
Displacement ellipsoid plot of the title molecule with the labelling scheme for the non-H atoms, which are drawn as 35% probability ellipsoids.

## supplementary crystallographic information

### Comment

Benzimidazole derivatives are known to possess a variety of biological properties (Le *et al.*, 2004), the anti-cancer activity being one of the most important (Nguyen *et al.*, 2004). Recently, it has been reported that the cytotoxic activity of 1*H*-benzimidazoles is related to inhibition of the DNA-topoisomerase binary complex and is potentiated by introduction to the 6-position of a small substituent containing an oxygen atom able to accept a hydrogen bond (*e.g.* nitro, acetyl, amide) (Alpan *et al.*, 2007; Statkova-Abeghe *et al.*, 2005). It was, however, unclear whether the influence of the substituents reflects their effect on the charge distribution of the heterocycle or results from interaction of the substituents with additional DNA or enzyme functionalities. Consequently, we prepared a series of 6-substituted 1-methylbenzimidazoles and determined and compared their molecular and electronic structures by using theoretical and experimental techniques. In this communication we report the crystal structure of the 6-nitro derivative, (I). Another point of interest in (I) stems from its use in paper processing as an antioxidant agent (Katuščák, 2003; Hanus *et al.*, 2004), a property which is also dependent on the molecular and electronic structures of the compound.

As expected, the 1-methylbenzimidazole substructure is planar to within experimental error and the nitro group is rotated by 10.4 (2)° from the plane of the heterocycle (Fig.1).

As mentioned above, the main purpose of this work was to compare precise molecular dimensions in the present derivative, (I), with those of the unsubstituted 1-methylbenzimidazole. As the latter compound has no entry in the Cambridge Structural Database (CSD, Version of 2007; Allen & Kennard, 1983), the CSD was searched for compounds possessing the benzimidazole nucleus and just 1-substituent with methylene group in the α-position; 42 such compounds [hereafter (II)] were found. The comparison have shown that the corresponding bond lengths in the benzimidazole heterocycle in (I) and in the molecules of (II) are equal within the limits of experimental error. This, along with the single-bond character of C6—N4 (Adler *et al.*, 1976) indicates that the nitro group is deconjugated with the benzimidazole ring. This implies that the large difference in cytotoxic activities between (I) and (II) lies in the interaction of the 6-substituent with additional DNA intercalation component or enzyme amino acid residues which surrounds the intercalation site (Kettmann *et al.*, 2004). These results will serve as a basis for subsequent molecular-modelling studies of the DNA-enzyme-ligand interactions.

### Experimental

The synthesis of the title compound, (I), was described earlier (Ellis & Jones, 1974). In short, a solution of formaldehyde (4 g, 0.133 mol) in absolute ethanol (40 ml) was heated under reflux for 30 min with commercially available 4-nitro-1,2-phenylenediamine (7.1 g, 0.046 mol) and concentrated hydrochloric acid (3 ml). On basification with ammonia, (I) was obtained as yellow crystals (25% yield; m.p. 454–456 K).

### Refinement

H atoms were visible in difference maps but were placed in calculated positions and were refined isotropic (*U*_iso_ of the H atoms were set to 1.2 (1.5 for the methyl H atoms) times *U*_eq_ of the parent atom) using a riding model with C—H = 0.93 Å (CH_arom_) and 0.96 Å (CH_3_).

### Crystal data

**Table:**

C_8_H_7_N_3_O_2_	*D*_x_ = 1.470 Mg m^−^^3^
*M**_r_* = 177.17	Melting point: 455 K
Orthorhombic, *P**b**c**a*	Mo *K*α radiation λ = 0.71073 Å
Hall symbol: -P 2ac 2ab	Cell parameters from 20 reflections
*a* = 12.852 (3) Å	θ = 7–19º
*b* = 7.043 (2) Å	µ = 0.11 mm^−^^1^
*c* = 17.690 (4) Å	*T* = 296 (2) K
*V* = 1601.2 (7) Å^3^	Prism, yellow
*Z* = 8	0.30 × 0.20 × 0.15 mm
*F*_000_ = 736	

### Data collection

**Table:**

Siemens P4 diffractometer	*R*_int_ = 0.035
Radiation source: fine-focus sealed tube	θ_max_ = 30.0º
Monochromator: graphite	θ_min_ = 2.3º
*T* = 296(2) K	*h* = −1→18
ω/2θ scans	*k* = −1→9
Absorption correction: none	*l* = −24→1
3027 measured reflections	3 standard reflections
2325 independent reflections	every 97 reflections
1493 reflections with *I* > 2σ(*I*)	intensity decay: none

### Refinement

**Table:**

Refinement on *F*^2^	Secondary atom site location: difference Fourier map
Least-squares matrix: full	Hydrogen site location: inferred from neighbouring sites
*R*[*F*^2^ > 2σ(*F*^2^)] = 0.045	H-atom parameters constrained
*wR*(*F*^2^) = 0.130	*w* = 1/[σ^2^(*F*_o_^2^) + (0.0722*P*)^2^] where *P* = (*F*_o_^2^ + 2*F*_c_^2^)/3
*S* = 0.96	(Δ/σ)_max_ = 0.002
2325 reflections	Δρ_max_ = 0.18 e Å^−^^3^
119 parameters	Δρ_min_ = −0.15 e Å^−^^3^
Primary atom site location: structure-invariant direct methods	Extinction correction: none

### Special details

**Table:**

Geometry. All e.s.d.'s (except the e.s.d. in the dihedral angle between two l.s. planes) are estimated using the full covariance matrix. The cell e.s.d.'s are taken into account individually in the estimation of e.s.d.'s in distances, angles and torsion angles; correlations between e.s.d.'s in cell parameters are only used when they are defined by crystal symmetry. An approximate (isotropic) treatment of cell e.s.d.'s is used for estimating e.s.d.'s involving l.s. planes.
Refinement. Refinement of *F*^2^ against ALL reflections. The weighted *R*-factor *wR* and goodness of fit *S* are based on *F*^2^, conventional *R*-factors *R* are based on *F*, with *F* set to zero for negative *F*^2^. The threshold expression of *F*^2^ > σ(*F*^2^) is used only for calculating *R*-factors(gt) *etc*. and is not relevant to the choice of reflections for refinement. *R*-factors based on *F*^2^ are statistically about twice as large as those based on *F*, and *R*- factors based on ALL data will be even larger.

### Fractional atomic coordinates and isotropic or equivalent isotropic displacement parameters (Å^2^)

**Table:**

	*x*	*y*	*z*	*U*_iso_*/*U*_eq_	
N1	0.42194 (9)	0.26257 (16)	0.47003 (6)	0.0416 (3)	
C2	0.52515 (10)	0.2941 (2)	0.45973 (9)	0.0497 (4)	
H2	0.5525	0.3360	0.4140	0.060*	
N3	0.58338 (9)	0.26080 (19)	0.51910 (7)	0.0532 (3)	
C4	0.53023 (11)	0.1419 (2)	0.64798 (8)	0.0553 (4)	
H4	0.5968	0.1419	0.6686	0.066*	
C5	0.44659 (12)	0.0845 (2)	0.68983 (8)	0.0544 (4)	
H5	0.4561	0.0432	0.7393	0.065*	
C6	0.34599 (10)	0.08772 (19)	0.65813 (7)	0.0425 (3)	
C7	0.32568 (9)	0.14664 (19)	0.58497 (7)	0.0390 (3)	
H7	0.2587	0.1496	0.5650	0.047*	
C8	0.41167 (10)	0.20061 (17)	0.54376 (7)	0.0374 (3)	
C9	0.51357 (10)	0.20067 (19)	0.57335 (8)	0.0444 (3)	
C10	0.33948 (11)	0.2912 (3)	0.41540 (9)	0.0555 (4)	
H10A	0.3428	0.4185	0.3962	0.083*	
H10B	0.2733	0.2711	0.4393	0.083*	
H10C	0.3476	0.2029	0.3745	0.083*	
N4	0.25815 (11)	0.03037 (18)	0.70486 (7)	0.0499 (3)	
O1	0.16986 (9)	0.0586 (2)	0.68211 (6)	0.0653 (3)	
O2	0.27589 (11)	−0.0462 (2)	0.76606 (6)	0.0721 (4)	

### Atomic displacement parameters (Å^2^)

**Table:**

	*U*^11^	*U*^22^	*U*^33^	*U*^12^	*U*^13^	*U*^23^
N1	0.0348 (5)	0.0474 (6)	0.0425 (6)	−0.0030 (4)	−0.0002 (4)	0.0008 (5)
C2	0.0376 (7)	0.0532 (8)	0.0583 (8)	−0.0041 (6)	0.0085 (6)	−0.0036 (7)
N3	0.0334 (6)	0.0599 (8)	0.0663 (8)	−0.0019 (5)	0.0008 (5)	−0.0071 (6)
C4	0.0428 (7)	0.0662 (9)	0.0570 (8)	0.0084 (7)	−0.0178 (6)	−0.0072 (8)
C5	0.0592 (9)	0.0607 (9)	0.0434 (7)	0.0103 (7)	−0.0138 (7)	−0.0029 (7)
C6	0.0460 (7)	0.0436 (7)	0.0379 (6)	0.0027 (6)	−0.0017 (5)	−0.0048 (5)
C7	0.0350 (6)	0.0417 (6)	0.0404 (6)	0.0007 (5)	−0.0039 (5)	−0.0044 (6)
C8	0.0349 (6)	0.0376 (6)	0.0398 (6)	0.0011 (5)	−0.0036 (5)	−0.0046 (5)
C9	0.0348 (6)	0.0450 (7)	0.0535 (8)	0.0029 (5)	−0.0055 (6)	−0.0089 (6)
C10	0.0477 (8)	0.0748 (10)	0.0441 (7)	−0.0036 (7)	−0.0071 (6)	0.0084 (7)
N4	0.0605 (8)	0.0515 (7)	0.0377 (6)	−0.0001 (6)	0.0029 (5)	−0.0052 (5)
O1	0.0503 (6)	0.0928 (9)	0.0527 (6)	−0.0051 (6)	0.0055 (5)	0.0053 (6)
O2	0.0891 (10)	0.0851 (9)	0.0420 (6)	0.0051 (7)	0.0043 (6)	0.0149 (6)

### Geometric parameters (Å, °)

**Table:**

N1—C2	1.3571 (17)	C6—C7	1.3840 (19)
N1—C8	1.3817 (17)	C6—N4	1.4563 (18)
N1—C10	1.4483 (18)	C7—C8	1.3775 (18)
C2—N3	1.3107 (19)	C7—H7	0.9300
C2—H2	0.9300	C8—C9	1.4104 (17)
N3—C9	1.3803 (18)	C10—H10A	0.9600
C4—C5	1.366 (2)	C10—H10B	0.9600
C4—C9	1.4001 (19)	C10—H10C	0.9600
C4—H4	0.9300	N4—O1	1.2202 (16)
C5—C6	1.4095 (19)	N4—O2	1.2308 (16)
C5—H5	0.9300		
			
C2—N1—C8	105.76 (11)	C8—C7—H7	122.4
C2—N1—C10	127.08 (12)	C6—C7—H7	122.4
C8—N1—C10	127.15 (11)	C7—C8—N1	131.56 (12)
N3—C2—N1	114.93 (13)	C7—C8—C9	123.28 (12)
N3—C2—H2	122.5	N1—C8—C9	105.17 (11)
N1—C2—H2	122.5	N3—C9—C4	130.31 (13)
C2—N3—C9	103.93 (12)	N3—C9—C8	110.22 (12)
C5—C4—C9	118.57 (13)	C4—C9—C8	119.46 (13)
C5—C4—H4	120.7	N1—C10—H10A	109.5
C9—C4—H4	120.7	N1—C10—H10B	109.5
C4—C5—C6	120.08 (13)	H10A—C10—H10B	109.5
C4—C5—H5	120.0	N1—C10—H10C	109.5
C6—C5—H5	120.0	H10A—C10—H10C	109.5
C7—C6—C5	123.36 (13)	H10B—C10—H10C	109.5
C7—C6—N4	117.89 (12)	O1—N4—O2	122.26 (14)
C5—C6—N4	118.74 (12)	O1—N4—C6	119.23 (12)
C8—C7—C6	115.24 (11)	O2—N4—C6	118.50 (13)
			
C8—N1—C2—N3	−0.55 (16)	C10—N1—C8—C9	−178.72 (13)
C10—N1—C2—N3	178.49 (14)	C2—N3—C9—C4	178.82 (15)
N1—C2—N3—C9	0.52 (16)	C2—N3—C9—C8	−0.29 (15)
C9—C4—C5—C6	−0.9 (2)	C5—C4—C9—N3	−178.51 (14)
C4—C5—C6—C7	0.2 (2)	C5—C4—C9—C8	0.5 (2)
C4—C5—C6—N4	−178.40 (14)	C7—C8—C9—N3	179.82 (12)
C5—C6—C7—C8	0.9 (2)	N1—C8—C9—N3	−0.02 (15)
N4—C6—C7—C8	179.47 (11)	C7—C8—C9—C4	0.6 (2)
C6—C7—C8—N1	178.54 (13)	N1—C8—C9—C4	−179.25 (12)
C6—C7—C8—C9	−1.26 (19)	C7—C6—N4—O1	−9.28 (19)
C2—N1—C8—C7	−179.50 (14)	C5—C6—N4—O1	169.38 (13)
C10—N1—C8—C7	1.5 (2)	C7—C6—N4—O2	170.32 (13)
C2—N1—C8—C9	0.32 (14)	C5—C6—N4—O2	−11.02 (19)
